# CALENDAR

**DOI:** 10.1054/bjoc.1999.1135

**Published:** 2000-03-21


					59 April 2000
Eurogin 2000
Global Challenge of Cervical Cancer Prevention
4th International Multidisciplinary Congress
Paris, France
Further information from:
Congress Secretariat, BAXON, N. Duraincie, C Boussin,
220-224 boulevard Jean, Jaures, 92773 Boulogne Cedex, France.
Tel: + 33 (0) 15520 23 83; Fax: + 33 (0) 1552023 93;
E-mail: duraincie.baxon@covos.fr
1113 April 2000
MICRO 2000 - International Microscopy Conference &
Exhibition
London, UK
Further information from:
MICRO 2000, Royal Microscopical Society, 37/38 St Clements,
Oxford OX4 1AJ, UK. Tel: +44 (0) 1865 248768; Fax: +44 (0)
1865 791237; E-mail: jenny@rms.org.uk;
Webpage: www.rms.org.uk/mic2000.html
2324 June 2000
II Melanoma Meeting for Specialists in dermatology,
surgery and medical oncology
Milan, Italy
Further information from:
Scientific Secretariat, Dr Alessandro Testori, European Institute
of Oncology, via Ripamonti 435-20141 Milan, Italy. Tel : + 39 02
57489 493; Fax : + 39 02 57489878; E-mail : atestori@ieo.it
or Organizing Secretariat, M.A.F. Servizi Srl, via G.B. Vico 7,
10128 Torino, Italy. Tel : + 39 011/505900; Fax : + 39 011/505
976; E-mail: melanoma2000@mafservizi.it
27 September  1 October 2000
Pigment Cell research - Perspectives for the Third
Millenium
University of Ulm/Donau, Germany
Further information from:
Department of Dermatology, University of Ulm, ESPCR 2000,
89070 Ulm/Donau, Germany. Tel: + 49 (0) 731 50 22251;
Fax: + 49 (0) 731 50 23772; E-mail: ralf.peter@medizin.uni-
ulm.dei; Website: http://www.uni-ulm.de/klinik/dermatologie
2830 September 2000
3rd Congress of the German Society of Palliative
Medicine
Gottingen, Germany
Further information from:
Kongress - Sekretariat, Frau G. Albrecht, Zentrum
Anaesthesiologie, Rettungs - und Intensivmedizin oler Georg -
August - Universitat Gottingen, Robert - Koch - Strasse 40,
D - 37075 Gottingen, Germany. Tel : + 49 551 39 88 26;
Fax : + 49 551 39 86 76; E-mail : gahlbre @gwdg.de
1013 July 2000
29th British Congress of Obstetrics and Gynaecology
International Convention Centre
Birmingham, UK
Further information:
BCOG Secretariat, Congress House, 65 West Drive, Cheam,
Sutton, Surrey SM2 7NB, UK. Tel: + 44 (0) 20 8661 0877;
Fax: + 44 (0) 20 8661 9036; E-mail: info@conforg.com
CALENDAR
British Journal of Cancer (2000) 82(8), 1493
(c) 2000 Cancer Research Campaign
Article no. bjoc.1999.1135, available online at http://www.idealibrary.com on
1493

				

